# Application of human induced pluripotent stem cells for tissue modeling and therapy: are we on track?

**DOI:** 10.1093/stmcls/sxag027

**Published:** 2026-05-07

**Authors:** Mathew Nickel Maunu, Ingrid Meulenbelt, Yolande F M Ramos

**Affiliations:** Department of Biomedical Data Sciences, Section Molecular Epidemiology, Leiden University Medical Center (LUMC), Leiden, The Netherlands; Department of Biomedical Data Sciences, Section Molecular Epidemiology, Leiden University Medical Center (LUMC), Leiden, The Netherlands; Department of Biomedical Data Sciences, Section Molecular Epidemiology, Leiden University Medical Center (LUMC), Leiden, The Netherlands

**Keywords:** human-induced pluripotent stem cells, disease modeling; cellular heterogeneity, methodological standardization, artificial intelligence–based cell fate prediction

## Abstract

Stem cells are key for development of disease modeling and therapies. While promising, however, current application of cutting-edge hiPSC technologies is, among others, confounded by cellular heterogeneity leading to concerns about their suitability for experimental and clinical applications. Variations across donors, tissue sources, methodologies, and analytical challenges, together contribute to the observed heterogeneity. Hence, increased understanding of heterogeneity in stem cell research is essential to advance development of reliable tissue models and effective therapies. In this review, we summarize current knowledge regarding the origins of cellular heterogeneity in hiPSC-derivatives. Differentiation protocols can be improved through the application of novel media morphogens, integration with new biomaterials and physical strategies (eg, 3D culture, mechanical stimulation). Additionally, standardization of methods and regulations for generation and application of cell lines and neo-tissues, thorough characterization, central banking, and registration of cells will reduce variation and increase experimental reproducibility. As reliable reference datasets become more abundant the continuous development of analytical tools as well as advanced application of artificial intelligence to analyze omics datasets will become more refined. This will aid identification of different cell types in heterogeneous cell populations and key factors driving off-target differentiation. We provide recommendations for best practices throughout the stem cell research pipeline and discuss opportunities to advance broad applicability of stem cells for disease modeling and beyond through concerted efforts to improve experimental robustness and analytical accuracy. Finally, we advocate that certain heterogeneity may be essential in development of laboratory models to faithfully mimic the in vivo situation.

## Graphical Abstract

**Graphical Abstract:**
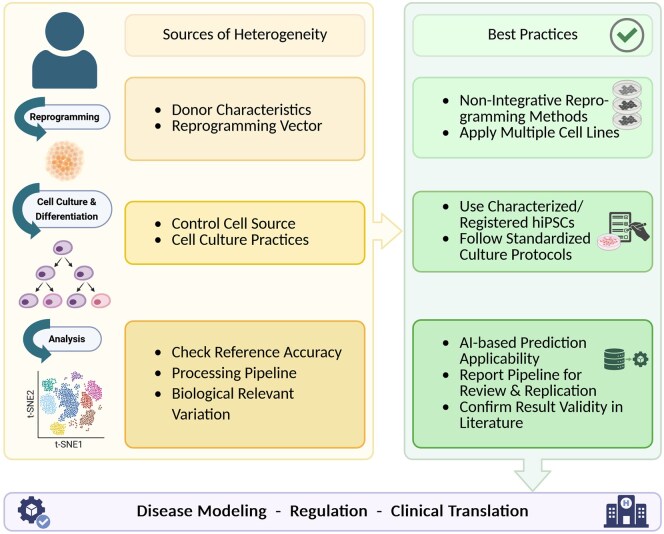

Significance statement Human iPSCs hold tremendous potential for the development of advanced tissue models and regenerative therapies. In this review we summarize current developments in the field and insights regarding the origins of unsolicited cellular heterogeneity in hiPSC cultures. We highlight methods for its detection and control, and discuss the potential biological relevance of this variability, where possible focusing on human studies published in the past 5 years. Based on this, we are convinced that the field is well on the way to improve reproducibility in the near future which will ensure improved disease modeling and development of therapeutics.

## Introduction

**Figure 1.**
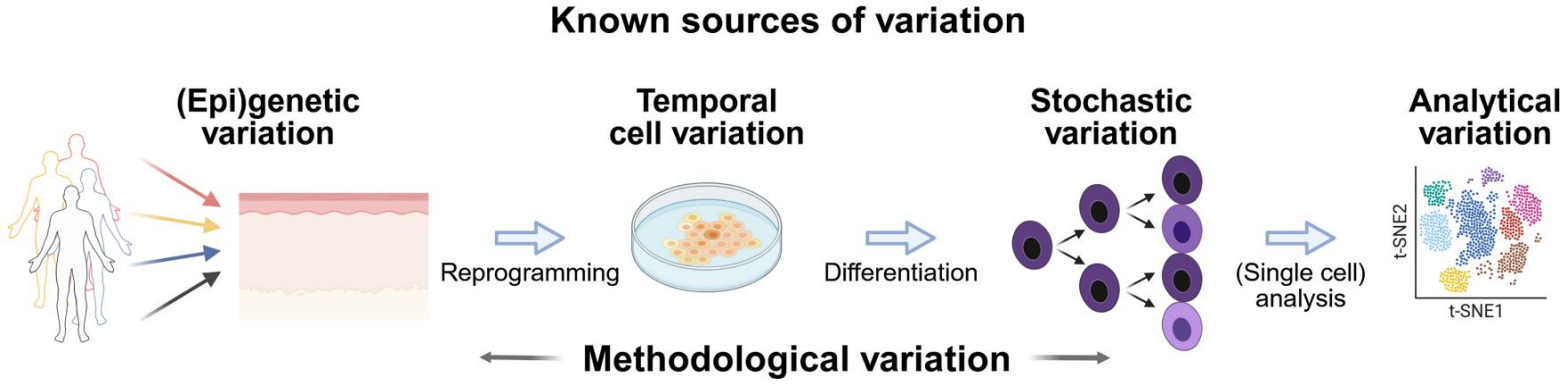
Schematic representation of known sources contributing to identified cellular heterogeneity. Over the course of experimentation population heterogeneity within hiPSC lines can originate from donor differences, methodological variation, and/or analytical tools and misreading of data.

Cell-based regenerative therapies and disease modeling platforms depend on accurate and consistent culture practices that faithfully recapitulate the characteristics of primary tissues. Recent advancements in stem cell-based systems, particularly those employing human induced pluripotent stem cells (hiPSCs), have substantially elevated both scientific and clinical expectations.^1^ For that matter, hiPSCs provide an ethically sustainable and highly versatile cell source capable of generating, on demand, diverse cell types in a human context for generation of relevant disease-associated tissues serving research or development of therapies. Application of hiPSCs, however, remains sensitive to experimental variability which introduces cellular heterogeneity and challenges experimental robustness.^2^ Variability arises from diversity in reprogramming techniques, subsequent culture and differentiation methods, inherent biological factors, and analysis methods (Figure 1). Moreover, the relatively immature nature of many hiPSC-derived tissues has raised concerns regarding their reliability in both tissue modeling and therapeutic contexts.^3^ Despite these challenges the advantages of hiPSC technology such as their potential to be maintained and expanded for longer periods of time and stored in cell banks, which contrasts with primary cells that have a limited cell culture window and frequently suffer from scarcity due to lack of donor availability, has strongly motivated ongoing efforts to identify and mitigate factors driving cellular heterogeneity. We here summarize current knowledge regarding the origins of cellular heterogeneity in hiPSCs drawing primarily on the framework drawn by Hayashi et al.,^4^ and highlight recent advances in methods for its detection and control through review of human studies published over the past 5-6 years. Importantly, we also discuss whether some heterogeneity may be biologically relevant to attain mature physiologically reliable tissues since, during in vivo development, differentiations are subject to cell–cell interactions with multiple cell types.

## Cellular heterogeneity

Heterogeneity in hiPSC lines and tissues derived therefrom arises from a combination of biological, technical, and analytical factors. Biological heterogeneity encompasses variation originating from the somatic tissue of origin, stochastic processes such as gene expression changes and interactions with the microenvironment. Technical heterogeneity, among others, refers to reprogramming and culture methods. Analytical heterogeneity results from variability introduced during data generation or processing. Sources and impact of heterogeneity have been addressed in previous reviews.^2^^,^^4^^,^^5^ We here add the latest insights and developments based on recent publications and provide an overview of validated protocols and analyses. Inevitably, various sources of variation may act synergistically thereby enhancing cellular heterogeneity both within and across hiPSC populations during maintenance and experimental handling.

### Generation and maintenance of hiPSCs

Reprogramming of primary cells for generation of hiPSCs typically involves induction of pluripotency markers OCT4, SOX2, KLF4, and c-MYC (the Yamanaka factors).^3^ Previously, reprogramming relied heavily on integrating retroviral or lentiviral vectors which insert randomly into the genome and can disrupt genes essential for normal cellular function. These uncontrolled integration events pose risks for experimental interpretation and limit translational potential of such lines. To address these concerns, integration-free methods have been developed. The most common non-integrating methods to date are adenoviral and Sendai viral delivery as well as episomal vector and protein-based transfection (Table 1). Notably, non-integrating methods typically show lower efficiency (10^−2^% to 10^−3^% for viral systems versus 10^−4^% to 10^−5^% for non-viral systems).^6^ As an alternative, application of chemical reprogramming methods based on small molecules have been explored more recently.^7^ Nevertheless, the cellular trajectory of different reprogramming approaches requires further research, among others to increase success rate and identify markers indicating correct progression of the reprogramming process which is particularly important for translational applications.

**Table 1:** Reprogramming methods and characteristics.

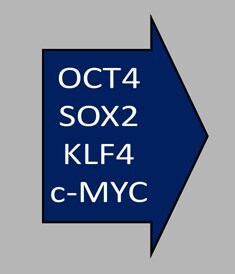	**Integrative** 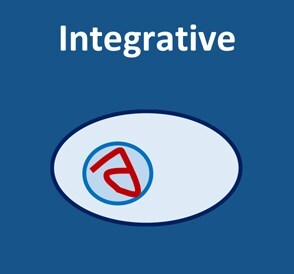	**Non-integrative** 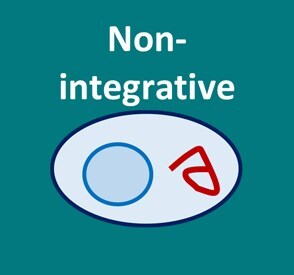	**Transfection** 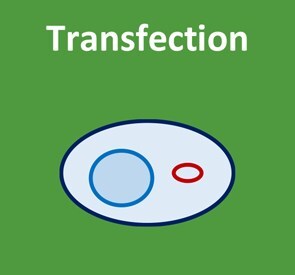	**Other** 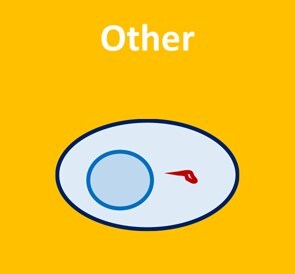
**Virus**	Retrovirus; lentivirus	Adenovirus; sendai virus	–	–
**Episomal**	–	–	Vector; mRNA technology; protein-based	Small molecules
**Integration**	++	–	–	–
**Efficiency**	+++	+	+/−	–
**Concerns**	Tumorigenicity	Costs; biosafety	Costs; labor-intensive	Low success rate

In this respect, RNA sequencing and DNA methylation analyses have demonstrated that epigenetic and chromatin modifications are not always fully reset during reprogramming, resulting in a partially reprogrammed state that subsequently affects differentiation efficacy.^1^ Genes contributing most to this variability are often involved in stem cell maintenance and lineage specification.^8^ In line with this, differential expression of *MIXL1* (Mix Paired-Like Homeobox, a transcription factor playing a central role in early development) was found to drive an endoderm formation bias during differentiation. Likewise, variants in *MIXL1*^9^ but also in *CER1*^10^ (Cerberus 1, a BMP antagonist) and in *BCOR*^11^ (BCL6 Corepressor) affect hiPSC growth rates hence increasing heterogeneity due to subclonal growth differences. Evidently, cellular heterogeneity within the tissue of origin will introduce unwanted variation into the hiPSC population.^5^

Beyond reprogramming, by comparing large numbers of hiPSC lines, up to 50% of experimental variability has been attributed to donor-specific genetic differences.^8^^,^^12^ Although the tissue of origin was thought to contribute significantly to variability across different hiPSC lines due to epigenetic memory of the cells, it has now been demonstrated that, upon differentiation, tissue-specific signatures are generally not maintained.^13^^,^^14^ Of note, in fibroblast-derived hiPSCs, the most common donor cell type, environmental factors such as ultraviolet-exposure of the donor can substantially increase accumulation of spontaneous mutations. While some variation between donors is expected and adds to the value of hiPSCs (eg, when performing so called “clinical trials in a dish”), variation should be consistent with the source of the material and not a result of experimental disparity and handling of the cells. Without thorough characterization, screening, and subcloning of hiPSCs, such heterogeneity will become increasingly pronounced upon long-term culturing due to varying clonal expansion rates, adding to variability resulting from donor age, ethnicity, and sex.^15^

To enhance reproducibility and translational reliability the International Society for Stem Cell Research (ISSCR) has provided guidelines outlining best practices for hiPSC maintenance and quality assessments toward standardization of common practice.^16^ These guidelines are regularly updated to address emerging scientific and ethical challenges. Recommendations include karyotyping and genetic screening for point mutations upon hiPSC-generation and during long-term expansion, flow cytometric analyses for surface markers and/or transcriptomic analysis for target genetic variants particularly following genetic manipulation of hiPSCs with technologies such as CRISPR/Cas9. For publicly available cell lines, information acquired during the characterization should appropriately be documented in public databases such as hPSCreg or Cellosaurus to ensure convenient access for users. Accordingly, several hiPSC repositories have been established that provide well-characterized, quality-controlled cell lines, for example, ATCC, CiRA, and EBiSC.^17^

In addition to the variation that is observed within hiPSC-populations and derivatives thereof, heterogeneity of results is also reliant on the researchers performing the experiments. By comparing transcriptomic profiles from hiPSC-derived cortical neurons from the same hiPSC lines and following the same protocols, the research team was shown to account for up to 60% of the variation across the results.^18^ Differences originated from site-specific practices which are often not disclosed in publications such as cell passage number before differentiation, media volume changes, feeding at weekends, and use of frozen progenitor cells as well as other uncovered factors that, together, masked almost all biological effects. However, upon applying normalization methods, related site-specific confounders can be adjusted for to reveal relevant biological signals including genotypic effects. Taken together, transparency, adhering to provided standards and access to established, consistent, well-characterized cell banks that can be drawn from over time for experimental use is critical to maintain the integrity of each cell line and promote reproducibility throughout the hiPSC research community. It will additionally enable multi-line treatment testing and sex comparison in therapy development. The most important requirement to accomplish this is teamwork and close collaboration among different research groups studying related research questions.

### *In situ* cellular variation

In vivo, stem cells are intrinsically heterogeneous due to their capacity to give rise to a multitude of specialized cell types required for fetal development and tissue repair.^19^^,^^20^ Spatial orientation within the developing cell mass together with intercellular signaling are key determinants for proper tissue and organ formation. The interplay of these factors contributes not only to developmental cell type variation but also to cell state variation within individual populations.^21^ Consequently, molecular profiling of cells with different states can misleadingly suggest heterogeneity within a population and result in false identification of new subtypes. Work from Mabrouk and colleagues, for example, demonstrated that, under standard in vitro culture conditions, hiPSCs exhibit spatial self-organization characterized by heterogeneous OCT4 expression.^22^ Transcriptional and proteomic variability were shown not to represent distinct cell types but rather reflect spatial and temporal variation within an otherwise homogeneous hiPSC colony. Furthermore, experimental processing itself can introduce artificial heterogeneity. Cells subjected to stress-inducing isolation procedures often display elevated expression of stress-response genes compared with more gently handled counterparts.^23^ This unintended variation can also lead to identification of apparent heterogeneity within a cell population.^21^ To minimize such artifacts, single-cell analyses should be performed on cells handled as minimally and gently as possible (eg, reduce pipetting, optimize buffers, choose gentle matrix removal solutions). Encapsulation of cells within (single cell) hydrogels that mimic the cellular 3D environment offers a promising approach that reduces the need for mechanical processing for analyses. It facilitates straightforward cell recovery and minimizes need to degrade the de novo deposited extracellular matrix.^24^^,^^25^ Furthermore, publications should thoroughly describe applied cell processing and analytical pipelines to allow accurate replication of the methods applied, in line with the need for transparency mentioned before.

### Analysis and cell type recognition

The increasing resolution of modern analytical technologies together with the development of advanced annotation tools for diverse datasets including single-cell RNA sequencing (scRNAseq), whole-exome sequencing, and methylome profiling has revealed substantial variability within cell populations generated through hiPSC differentiation, frequently referred to as off-target cells.^26–28^ While such findings have raised concerns regarding the accuracy of hiPSC-based studies and their safety for clinical applications,^2^^,^^3^ it is important to recognize that part of this apparent heterogeneity may reflect genuine biological diversity as we will discuss later, but can also arise from inconsistencies in analytical methodologies rather than being true discrepancies in cell identity. The latter is what we address here following.

Annotation of cell types frequently relies on limited sets of marker genes derived from incomplete or poorly matched reference datasets. This can lead to misclassification, particularly when reference materials are themselves heterogeneous or derived from unrelated biological contexts. Identifying a robust and representative “golden standard” for cell type annotation is inherently difficult, as many cell populations remain insufficiently characterized and variation persists between laboratories and experimental setups. The challenge is compounded by the rapid evolution of analytical technologies, which continuously reshapes the landscape of available reference data.^29^ In the context of scRNA-seq, cell identity may depend on the differential expression of a relatively small subset of genes that can vary significantly among cells within a population.^30^^,^^31^ When such marker genes are not strictly cell-type specific or when bias is introduced during sample preparation or data processing, small differences in analytical thresholds can shift the detection of key genes, thereby altering cell clustering and classification for large portions of the dataset.^32^ Nevertheless, as single-cell multi-omics approaches such as combined ATAC-seq and RNAseq become increasingly available, the quality and interpretability of reference datasets are expected to improve.^33^ These multimodal datasets provide complementary layers of information that enhance the resolution of cell type identification and reduce ambiguity caused by transcriptomic noise. Until approaches such as multi-modal data analyses become routine and reference datasets are more thoroughly characterized and standardized, transparency in data annotation, analysis, and interpretation remains essential to allow validation and replication. Researchers bear the responsibility of implementing rigorous quality control measures, openly sharing analytical pipelines, and critically evaluating both their own results and those of others to ensure experimental robustness and reproducibility.

## Controlling heterogeneity

In contrast to most primary cells which typically senesce after only a few weeks and within ten passages, a key advantage of hiPSCs is their ability to self-renew and remain stable throughout numerous passages. This extended culture capacity allows for large-scale cell expansion and greater experimental flexibility. However, long-term expansion also poses risks and mutations as well as population heterogeneity accumulated over successive passages can compromise genomic and cell line stability. Hence, quality control measures to ensure continued population purity should be in place during continued hiPSC culture to confidently achieve reproducible and lineage-specific outcomes. Several complementary strategies have been developed to address this challenge during differentiation, including the use of specific culture additives, manipulation of environmental cues, and application of mechanical or material-based stimuli.

### Controlling cell fate through media additives

**Figure 2.**
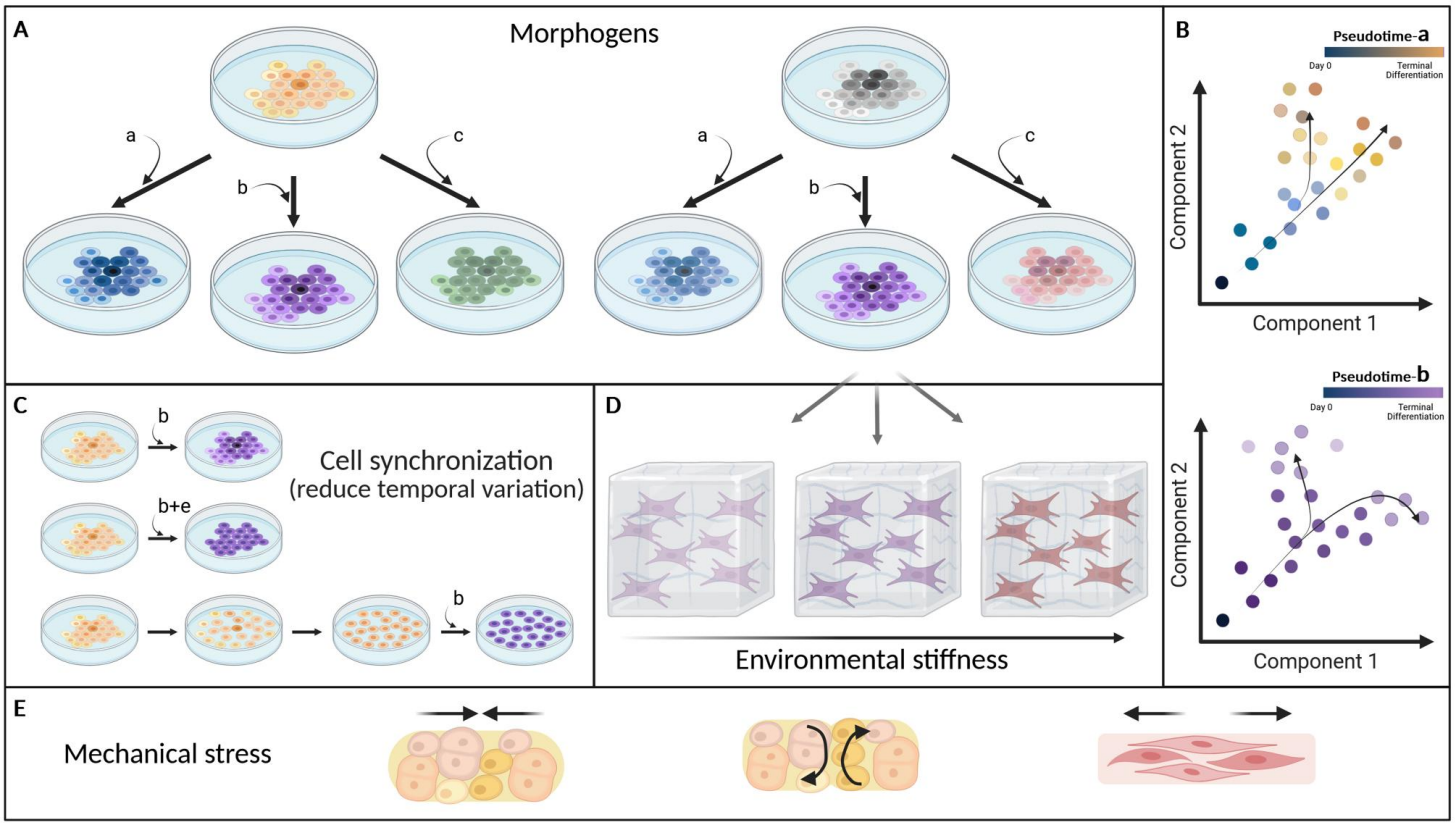
Schematic representation of cues that contribute to control cellular heterogeneity. (A) Varying morphogens and tissue sources impact hiPSC cell fate determination. (B) Pseudotime of hiPSCs through differentiation can be resolved using Monocle and shows variable levels of heterogeneity depending on media additives. (C) Limitation of cell fate variation due to cell state variation via cell synchronization through additives and/or cell passaging. (D) Environmental cues (eg, stiffness) and (E) mechanical stress (eg, load, shear, or stretch) can steer cell fate decisions.

In vitro differentiation protocols are designed to replicate developmental signaling and gene expression dynamics observed in vivo. By emulating these pathways, extended developmental timelines can be condensed, and cell state transitions can be more precisely controlled to yield the desired cell type. The most common and effective approach to directing hiPSC differentiation involves the addition of defined culture supplements, often referred to as morphogens (Figure 2A).^34^^,^^35^ These additives modulate cell fate by activating or inhibiting specific signaling pathways that induce commitment toward a desired lineage. Importantly, both the timing and concentration of these factors are critical, as the same morphogen can produce divergent outcomes depending on the differentiation stage or context. To illustrate, for generation of cartilage^26^^,^^36^ and cardiac^37^ organoids as well as for peripheral blood derivatives^38^ both CHIR-99021 (a glycogen synthase kinase 3 inhibitor) and BMP4 (bone morphogenetic protein 4) are used, yet the timing and concentration of application determines the final tissue. Identifying hub genes and regulatory nodes along differentiation trajectories can greatly advance accuracy of targeted approaches and further optimize differentiations with fitting and timely factors. Moreover, it will allow to follow lineage-specific changes hence indicate on- or off-target progress. This can be achieved through approaches such as longitudinal (single-cell) RNAseq and integrative omics analyses (Figure 2B) and was in fact successfully applied to improve hiPSC-derived chondrogenesis with C59-mediated inhibition of WNT4 and WNT2B signaling upon recognition of their strong association with off-target cells.^26^ Finally, synchronization of cell populations prior to differentiation can also contribute to minimize state-dependent differences and increase population homogeneity. This can be done either mechanically (eg, single-cell dissociation) or chemically (Figure 2C). The latter was effectively applied to improve hiPSC-derived pancreatic progenitors upon cell synchronization with botulinum hemagglutinin, an E-cadherin function-blocking agent that induces a synchronous switch between E- and N-cadherin expression which improved the capability for directed differentiation.^39^

### Added value of environmental cues

In addition to soluble morphogens and compounds, the physical and biochemical properties of the cellular microenvironment exert effects on hiPSC fate. Environmental parameters such as oxygen tension, spatial organization (2D versus 3D culture), and substrate composition including bioactive moieties that mimic cell–cell and cell–matrix interactions can influence differentiation efficiency and lineage commitment.^40–42^ By comparing 2D and 3D culture conditions, among others Rössler et al. demonstrated that hiPSC-derived 3D embryoid bodies differentiated into monocyte-like cells exhibited enhanced maturation relative to their 2D counterparts.^43^ Culturing in 3D allows cells to deposit and organize their own extracellular matrix, more closely resembling in vivo conditions. Despite these advantages, 3D cultures also introduce challenges for downstream analyses. Cell isolation from dense matrices often induces cellular stress as discussed before, potentially introducing bias in data output which could complicate subsequent cell-type classification.^23^ While mitigation strategies such as processing samples on ice and performing immediate post-isolation library preparations for sequencing can reduce these artifacts, further methodological refinement is required to fully eliminate and/or adjust for such biases.^44^ Possibly, application of multicellular 3D constructs including hydrogel-based scaffolds can be used to allow self-organization and to mimic the structural and mechanical features of native tissue environments^45^ while facilitating the collection of cells at set timepoints for analyses. Depending on the substrate material properties, including stiffness and biochemical functionality, this may also contribute to modulation, and advance stem cell stability and differentiation potential (Figure 2D). In this respect, Kamperman et al. demonstrated that microgel stiffness contributes to direct lineage specification of single encapsulated cells, with softer hydrogels favoring adipogenic differentiation and stiffer matrices promoting osteogenic outcomes.^24^

Differentiation of hiPSCs is an inherently delicate process that demands a deep understanding of mechanisms determining cell fate for directed optimization of conditions guiding these transitions. Applying bioactive substrates and environmental modulations such as matrix stiffness (Figure 2D) and mechanical stimulation facilitated by 3D culturing (Figure 2E) presents a versatile toolkit for controlling hiPSC differentiation and offers opportunities for improved efficiency, precision, and reproducibility in generating desired cell types. Mechanical stimuli such as compression or cyclic strain^46^ which have been shown to enhance differentiation into specific lineages such as chondrogenesis and osteogenesis as reflected by increased expression of lineage-specific markers.^47^^,^^48^ Naturally, each target cell type for hiPSC differentiation will require in-depth analysis of in vivo developmental conditions to serve as a reference, followed by generation of defined 2D or 3D environments depending on desired application and tissue type. Subsequently, integration into or development of complimentary morphogen protocols can be completed if desired.

## Mapping differentiation processes and analytical heterogeneity

Advances in computational methods such as omics analysis, artificial intelligence (AI), and mathematical modeling are being applied to analyze hiPSC differentiation. Along potential for methodological improvements^26^ it has provided a growing set of digital tools that reduce analytical heterogeneity while enabling correction for biologically irrelevant variability such as differences in transient cell states. Enhancing quality-control pipelines has been a critical first step in tailoring computational analysis programs to better account for both technological heterogeneity (eg, biased reference datasets, inaccurate thresholding of key genes, and batch effects) and biological heterogeneity (eg, differences among cell types, states, and donors).^23^^,^^44^ As single-cell sequencing technologies become more cost-effective and widely accessible, cutting-edge approaches such as multi-omics and spatial transcriptomics are emerging as powerful tools to dissect regulatory mechanisms that govern cell fate decisions.^30^^,^^49^^,^^50^ In parallel, the rise of advanced 3D culture systems including engineered tissue constructs, biomaterial scaffolds, and organ-on-chip models has increased interest in single-cell, multi-modal, and particularly spatial-omics analyses to assess cellular variation within physiologically relevant contexts.^51^ Additionally, imaging mass cytometry for spatial proteomics is available to characterize multiple tissue samples based on protein panels while preserving their microenvironmental context.^52^ Together, such techniques allow for the improved characterization of samples in important environmental and developmental contexts, progressing our understanding of differentiation and cellular processes.

Following cell-type identification, computational clustering algorithms can be integrated into developmental pipelines to further classify cells from single-cell datasets according to their similarity across transcriptomic, epigenomic, morphologic, or proteomic parameters.^51^^,^^53^^,^^54^ Applications such as the Monocle3 toolkit can be used to generate pseudotime analysis and infer continuous trajectories of cellular differentiation, proliferation, or oncogenic transformation.^55^ The algorithm assumes a gradual transition between cell states, arranging cells along a trajectory that begins with a basal state (eg, undifferentiated hiPSCs) and extends toward predicted terminal phenotypes as defined by distinct molecular profiles. Such analyses contribute to distinguish cell population purity and developmental maturity within dynamic systems across timepoints and allow to generate transitional datasets that capture intermediate cell states to resolve intra- and inter-sample heterogeneity and visualize relationships among identified cell populations. Notably, “RNAge” was introduced in 2025, a transcriptome-based computational platform that enables evaluation of cellular aging for the analysis of age-related manipulations.^56^ This type of technique could be useful in identifying methods for improving fidelity of age-related disease modeling using hiPSC-derived organoids and identifying key targets to improve hiPSC-derived neo-tissue maturity, hence similarity to autologous tissues.

### Non-destructive analysis approaches

**Figure 3.**
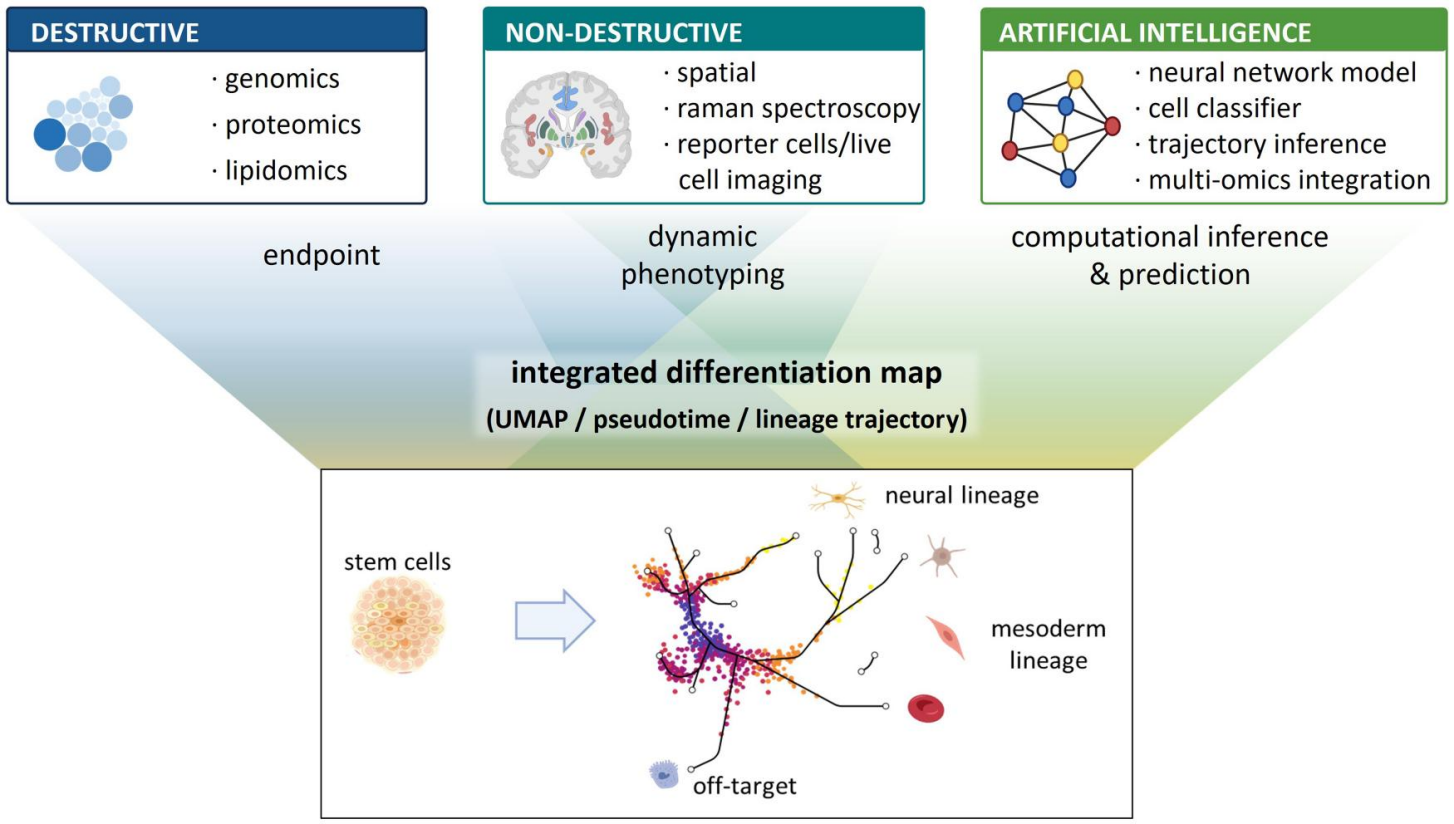
Analytical approaches for mapping of differentiation processes and neo-tissue formation. Datasets from tissues can be generated by applying destructive or non-destructive analysis techniques, with the latter allowing both *in situ* localization of specific targets as well as longitudinal data analysis, depending on the approach. Notably, to date non-destructive methods are limited with respect to number of targets. Processing and training of datasets with AI-models can contribute to predict and improve experimental outcomes thereby leveraging the impact of stem cell applications.

While single-cell analyses offer unparalleled resolution, common applications of these methods are destructive which limits its use for longitudinal studies (Figure 3). Non-destructive live-cell imaging approaches can be employed to monitor differentiation dynamics in real time. To this end fluorescent reporter cell lines in which a fluorophore is co-expressed with a gene of interest are widely used. This enables the quantification of gene expression dynamics and identification of off-target differentiation within a population and even allows fluorescence-activated cell sorting (FACS) to select specific subpopulations to increase cell culture homogeneity. As an example for use of reporter cell lines, the quality of engineered neocartilage organoids could be improved by sorting COL2-GFP-positive cells.^36^ Likewise, osteoblast differentiations from hiPSCs could be optimized by tracking expression levels of COL1A1 with a reporter line,^57^ and functional hiPSC-derived atrial cardiomyocytes could be distinguished from ventricular cardiomyocytes by selecting for lower or higher CD151 expression levels.^31^ On another note, it was shown that the expression level of CHD7 (Chromodomain Helicase DNA-binding protein 7) serves as a good prediction marker to monitor the divergence in differentiation potential within a population and to monitor differentiation initiation and progress.^58^ This information was used to optimize hiPSC culture conditions and develop insights into cellular and tissue developmental pathways. Given the function of CHD7 in early development this aligns well with earlier mentioned connection of stem cell maintenance and lineage specification genes contributing to cell population variability.

Other non-destructive methods such as Raman spectroscopy can complement transcriptomic approaches. It was used by Fujita et al. to monitor hiPSC heterogeneity in transitions during cardiomyocyte differentiation.^59^ Cellular heterogeneity was quantitated by spectroscopy and specific spectral peaks, most likely attributed to glycogen, were found to increase more rapidly than signal intensity changes upon loss of pluripotency which the authors explained to be useful as a biomarker for prediction of future cell state transitions. A more recent innovation, Live-seq, provides a non-destructive approach to single-cell transcriptomics. This technique, developed by the Vorholt laboratory, samples cytoplasmic material from living cells to reconstruct single-cell transcriptomes while preserving viability and functionality.^60^^,^^61^ Although currently limited by technical complexity and scalability, Live-seq and similar methods hold great promise for longitudinal tracking of single cell fate transitions during differentiation. As alternative non-destructive techniques such as Live-seq become more established, they will play an essential role in resolving the intricate dynamics of cellular heterogeneity in both in vitro and in vivo systems with less uncertainty than standard experimental practices.

### Application of artificial intelligence

Currently, the integration of AI into omics data analysis represents one of the most rapidly advancing areas in bioinformatics. AI-driven tools, including large language models (LLMs) for automated cell-type annotation and deep learning algorithms for clustering and fate prediction offer substantial improvements in analytical speed and accuracy compared with traditional pipelines.^62–64^ Many of these tools are now distributed as open-access R packages, facilitating broad implementation across different laboratories. Dobner and colleagues studied the effect of partial reprogramming of somatic cells as source for population heterogeneity by performing long-read nanopore transcriptome sequencing. In this way, the authors uncovered 172 genes linked to cell state (eg, pluripotency markers such as *NANOG*, and endoderm markers such as *CER1* and *GATA6*).^65^ The data was subsequently used to develop a machine learning-based scoring system named “hiPSCore” that can be used to classify pluripotent cells and predict their potential to become specialized 2D cells and/or 3D organoids. While the promise and broad applicability in analysis of AI is large, AI methods and LLM-based tools have been recorded to produce misleading or incorrect annotations, particularly when trained on incomplete or biased datasets, so called “hallucinations.” Moreover, method development requires extensive computational resources and heavily relies on the availability of robust historical datasets for model training. There is also a concern that generated outputs are frequently resulting from black-box processing where underlying algorithms are unknown to the user. Therefore, it is advised that researchers who apply novel AI technologies also perform traditional analyses in parallel or rely on confirmatory historical data to substantiate the AI outputs and educate themselves of the limitations regarding the specific techniques that they are applying.^62^ In any case, as AI continues to evolve its integration with high-resolution multi-omic data is expected to advance in precision, reproducibility, and interpretability also in hiPSC research.^63^

## Beneficial heterogeneity and clinical translation

To ensure the integrity of scientific outcomes and safety in clinical testing, it is critical that researchers can be confident that experimental conditions can be controlled. Therefore, ensuring high quality, homogenous cell sources and repeatable conditions is critical. In conjunction with this, early *in vivo* development is characterized by diverging cells committing to different lineages and giving rise to multiple tissues and organs that constitute the developing fetus. As discussed before, besides heterogeneity, a common challenge with hiPSC-derived tissues is their immaturity. This is also illustrated in Table 2 that lists established differentiation protocols and includes information about similarity to autologous tissues where available (see column “Similarity”). Studies have been performed where hiPSC-derived organoids have demonstrated increased functionality and cellular maturation upon transplantation. For example, this was shown for nephron sheets^95^ and pancreatic β cells.^86^ For the latter it was also shown that during development of the pancreas both in humans and in mice the initially observed cellular heterogeneity reduced over time.^96^ This could point either at a maturational catch-up of cells that were not yet at the stage of their surrounding cells or at the loss of off-target cells during in vivo organoid-maturation, for example, due to apoptosis. Furthermore, it has been demonstrated that some differentiations benefit from presence of other cell types. During differentiation of hiPSC toward sensory neurons the presence of Schwann cells was found to support efficiency of the neurogenesis.^97^ In this line, it should be considered that during hiPSC differentiation, in addition to off-target cells, also support cells are generated that incite formation and maturation of specific tissue-organoids. We advocate that, henceforth, classifications for differentiating hiPSCs and their heterogeneous profiles should be reconsidered and the value and risk of heterogeneity further established.

**Table 2:** Differentiation protocols for hiPSC-derived vital and non-vital organs.

		Tissue	References	Duration	Year	Cit.	(WoS)	Key techniques	Similarity
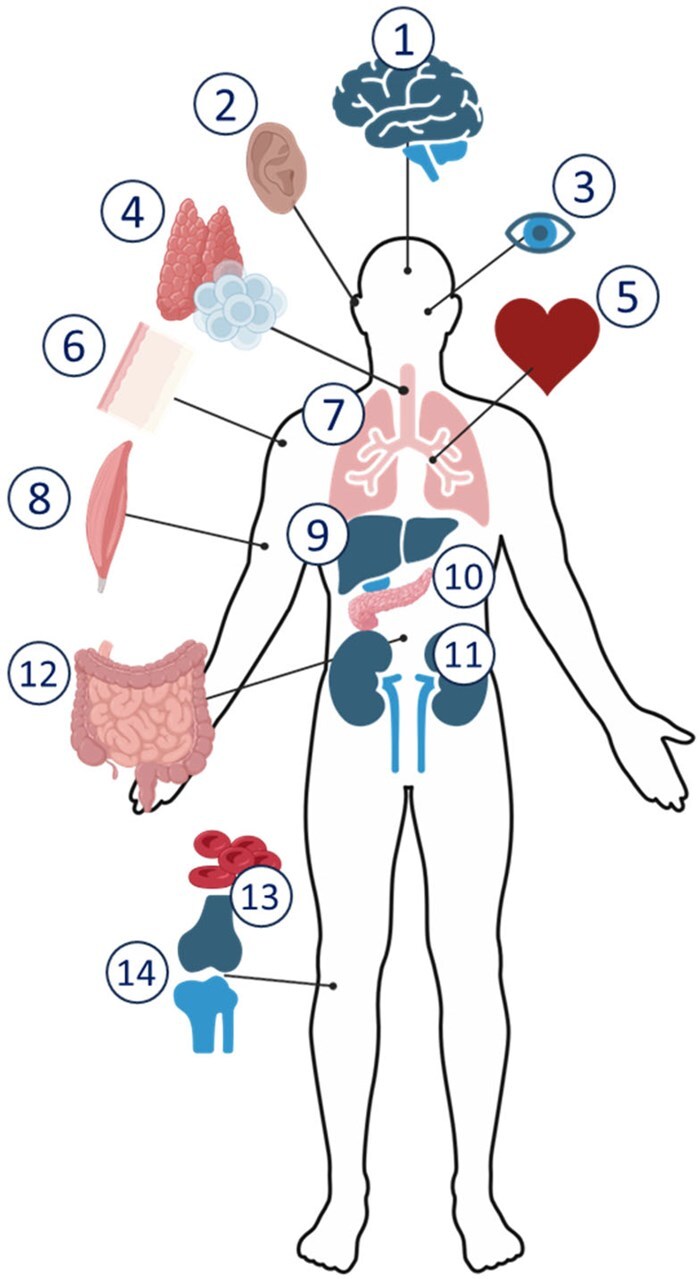	1	(Advanced) Cerebral organoids	Giandomenico et al.^66^	140 days	2020	272	(177)	^a^ ^,^ ^c^ ^,^ ^d^ ^,^ ^e^	NA
			Lancaster and Knoblich^67^	30 days	2014	1902	(1192)	^a^ ^,^ ^d^ ^,^ ^e^	NA
	2	Inner ear organoid	van der Valk et al.^68^	60-90 days	2025	0	0	^a^ ^,^ ^c^ ^,^ ^e^	NA
			van der Valk et al.^69^	>65 days	2023	9	0	^a^ ^,^ ^d^ ^,^ ^e^ ^,^ ^f^	NA
	3	Forebrain organoid with optic vesicles	Gabriel et al.^70^	60 days	2023	19	(13)	^a^ ^,^ ^e^	NA
			Gabriel et al.^71^	60 days	2021	160	(94)	^a^ ^,^ ^e^ ^,^ ^f^	NA
		Retinal pigmented epithelium	Surendran et al.^72^	55-75 day	2022	10	(0)	^a^ ^,^ ^e^	NA
			Surendran et al.^73^		2021	45	(30)	^a^	hiPSCs
	4	Thymus	Provin and Giraud^74^	21-30 days	2022	17	(12)	^a^ ^,^ ^c^ ^,^ ^d^ ^,^ ^e^ ^,^ ^f^	Other (different) protocols
		T cells	Iriguchi et al.^75^	14 days	2021	211	(152)	^a^ ^,^ ^e^	NA
	5	Heart-forming organoids	Campostrini et al.^37^	21 days	2021	118	(78)	^a^ ^,^ ^e^ ^,^ ^g^	NA
			Drakhlis et al.^76^	19 days	2021	51	(38)	^a^ ^,^ ^d^ ^,^ ^e^ ^,^ ^f^	Recently developed advanced model named blood-generating (BG)-HFO^77^; related scRNAseq by Dardano et al.^78^ suggests 75% of cells are still immature
	6	Skin	Lee et al.^79^	>130 days	2022	103	(72)	^a^ ^,^ ^e^	NA
	7	Lung organoid	Leko et al.^80^	>25 days	2023	50	(30)	^a^ ^,^ ^b^ ^,^ ^d^ ^,^ ^e^ ^,^ ^f^	NA
			Miller et al.^81^	22 days	2019	441	(313)	^a^ ^,^ ^c^ ^,^ ^d^ ^,^ ^e^	Native bud tip progenitors in human fetal lung (<16 weeks of gestation)
	8	Muscle	Akar et al.^82^	13-16 days	2024	8	(5)	^a^ ^,^ ^e^	NA
			van der Wal et al.^83^	13-16 days	2018	106	(83)	^a^	NA
	9	Liver buds	Takebe et al.^84^	4 days (plus ∼30)	2014	452	(286)	^a^ ^,^ ^e^	More alike to mouse E10.5-E11.5 liver buds than human fetal or adult livers
	10	Pancreatic β cells	Hogrebe et al.^85^	∼30 days	2021	200	(130)	^a^ ^,^ ^e^	Transcriptionally and functionally immature compared to native adult β cells (scRNAseq in Augsornworawat et al.^86^)
			Kim et al.^39^	13 days	2023	0	(0)	^a^	NA
	11	Proximal tubule-enhanced kidney organoids	Vanslambrouck et al.^87^	27 days	2023	20	(15)	^a^ ^,^ ^e^ ^,^ ^f^	Other (different) protocols: improved maturity
			Vanslambrouck et al.^88^	27 days	2022	80	(55)	^a^ ^,^ ^c^ ^,^ ^e^ ^,^ ^f^	Equivalent to E14.5 mouse kidney (bulk RNAseq in Takasato^89^)
		Kidney organoids	Wu et al.^90^	26 days	2018	625	(421)	^a^ ^,^ ^e^ ^,^ ^f^	Benchmarked against adult human single-cell datasets
	12	Intestinal tissue	McCracken et al.^91^	28-35 days	2011	528	(343)	^a^ ^,^ ^d^ ^,^ ^e^	NA
	13	Bone marrow organoids	Olijnik et al.^92^	>18 days	2024	40	(24)	^a^ ^,^ ^b^ ^,^ ^c^ ^,^ ^d^ ^,^ ^e^	NA
	14	Cartilage organoids	Dicks et al.^93^	42-49 days	2023	7	0	^a^ ^,^ ^b^ ^,^ ^e^ ^,^ ^f^	NA
			Hajmousa et al.^14^	42-49 days	2024	3	(2)	^a^ ^,^ ^b^ ^,^ ^e^ ^,^ ^f^	85% similarity to transcriptome and methylome profiles human chondrocytes

List of established protocols that include detailed description of experimental design, critical steps, and limitations with, where possible, an indication of similarity to autologous tissues. See also Haniffa et al. for a roadmap concerning human developmental cell atlas.^94^ Abbreviations: Cit: Pubmed citations; Duration: duration of the differentiation protocol; NA: not applicable; WoS: Web of Science citations.

a Reference involves addition of media morphogens.

b Reference manipulates oxygen concentrations.

c Reference involves the use of reporter cell lines.

d Reference involves the use of cellular scaffolds.

e Reference involves the use of multiple cellular culture orientations (2D, 3D, embryoid bodies/organoids, etc.).

f Reference involves the use of single cell omics technologies and advanced analytical methods.

g Reference involves the use of action potential recordings to demonstrate functionality.

Clinical trials require extensive documentation of the cell source, their handling and storage, strict quality controls for all products involved in the production of cell products, and long term safety and efficacy for the specific therapy. Once cell culture methods are standardized and best practices established, some hurdles for the translation of research outcomes into clinical products remain. For that matter, international research and clinical implementation is obstructed by the regulatory variation depending on the governing body. In the European Union (EU), regulations are managed by the EU parliament and commission through the European Medicines Agency (EMA, 2025).^98^^,^^99^ Regulations have also been outlined in other major landscapes for clinical development such as China (Measures for Ethical Review of Science and Technology, 2023), Japan (PMD Act Amendment, Act on the Safety of Regenerative Medicine [ASRM] 2014), and the USA (2016, 21st Century Cares Act). Additionally, there are still developments needed to make industrial scaling of stem cell processes economically viable, as media component costs remain high and stem cell treatments would require government subsidization to be accessible for the public. An example is CART therapy, where treatments can cost over $373 000 USD.^100^ Encouragingly, hiPSC-based trials that have been completed within regulatory guidelines did not show negative outcomes resulting from stem cell treatments,^101^ providing an important foundation for future trials. Considering the rapid adoption of related cellular therapies such as the CART treatment, it is apparent that the demand and opportunity to implement advanced cellular therapies is high.

## Conclusions and future perspective

**Figure 4.**
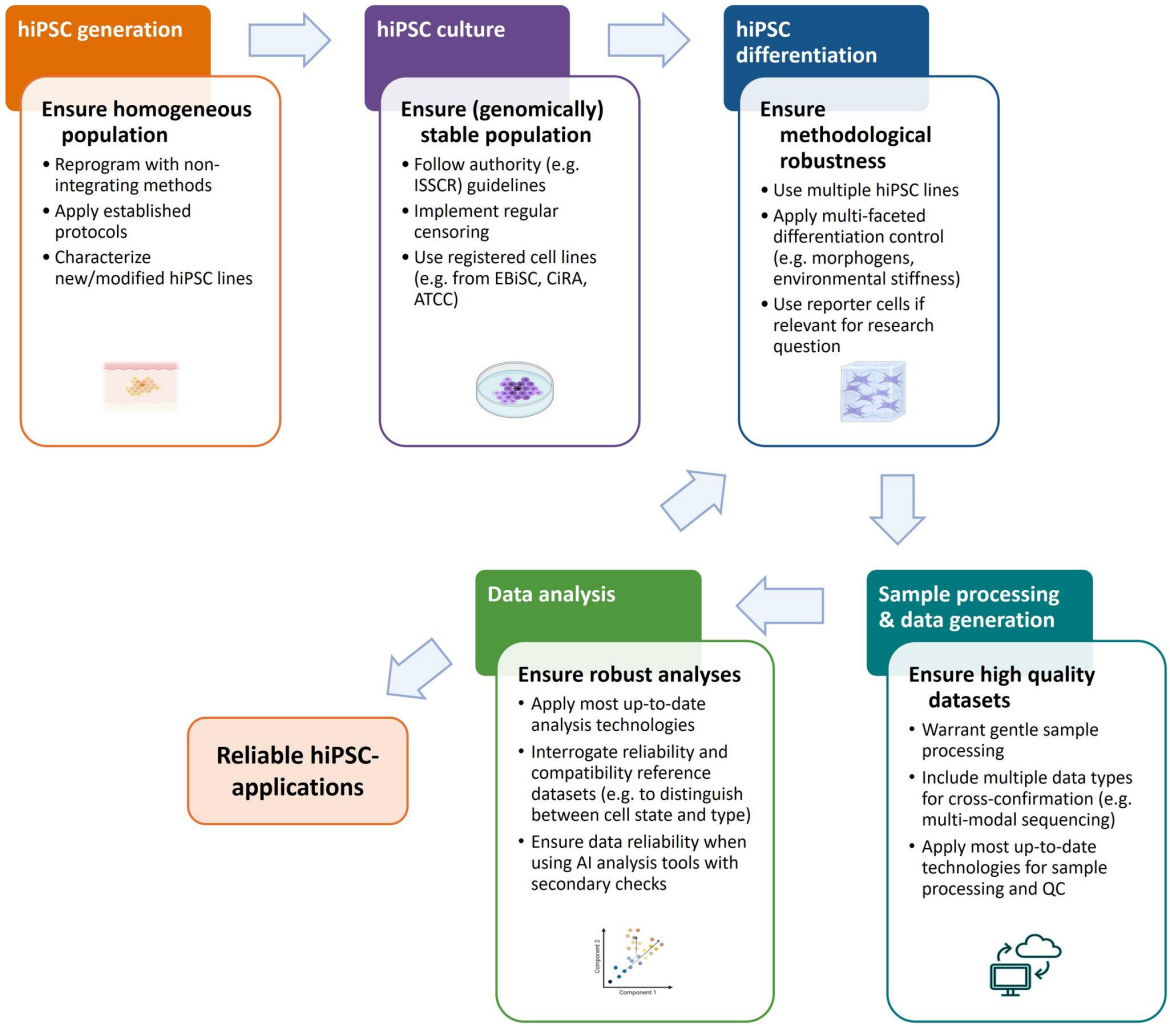
Outline of recommendations toward advanced hiPSC applications. The flowchart summarizes recommendations for best practices. Starting with hiPSC generation from donor tissues, their expansion, banking, quality control, through differentiations. Sample processing and data analysis can subsequently be used to improve prior steps via a positive feedback loop. At each step, utilization of recommended best practices will improve experimental reliability and reproducibility while improving the overall research efficiency.

Human iPSCs hold tremendous potential for the development of advanced tissue models and regenerative therapies and are continuously advancing for a wide range of tissues (Table 2). In 2016, Li and Belmonte in their perspective on “10 years of Nature Protocols” posed a call for increased basic research and closer collaboration with clinicians and policy makers to communicate the advantages of hiPSCs in clinical applications. Although challenges remain, continued international efforts toward methodological standardization in hiPSC culture and analysis tied to the rapid evolution of analytical and bioinformatics tools, have and are expected to significantly advance the field. Our recommendations to take this further on are outlined in Figure 4. It is important that researchers continue to adhere to the most updated cell culture practice guidelines of field authority organizations such as the ISSCR. With ongoing fundamental research, scientists should now connect with national and local regulatory authorities for guidance on requirements for transitioning to clinical trials and bring novel technologies to patients. In relation to therapy development there is a notable role for the EMA and national competent authorities (NCAs) to pave the way toward continued progress.^101^

In addition to adhering to provided guidelines and transparency, AI and newly developed LLM-tools should be further optimized and implemented to more precisely direct hiPS cell fate and concurrent reproducible outcomes. Such optimization may in due time lead to protocol changes regarding timing and application of specific culture additives, manipulation of environmental stimuli (eg, material stiffness, biochemical conjugates), and mechanical stimulation to refine lineage commitment. Notably, although spatial analyses are increasingly applied to autologous tissues, their adaptation to stem cell-based in vitro systems remains nascent and should be a point of attention in the coming years. Additionally, while there is still ample space for improvement of differentiation protocols and understanding of basic biological systems, for the field to progress there needs to be an increase in cross-disciplinary research. This would allow for integration of multiple technologies and creation of more effective solutions. Improvements to protocols from all fields will help improve hiPSC-derived cell maturity and similarity to in vivo tissues allowing more accurate cellular models and effective therapies.

Throughout academic and clinical development there must be uncompromising transparency in reporting of experimental approaches, including both positive and negative results. This will ensure improved experimental replication across different research teams and will ensure that scientists are efficiently moving toward clinical applications. Ultimately, a deeper understanding of the origins and potential biological relevance of cellular heterogeneity will further enable the development of strategies for its effective control. This progress will not only enhance the safety and clinical applicability of hiPSC-based therapies but also facilitate the generation of highly faithful cellular models that accurately recapitulate human tissue physiology. Altogether, we are convinced that the field is well on the way to establish robust methods for reproducible and clinically relevant results in the near future.

## Data Availability

Not applicable.
